# Frequency of Ophthalmological Findings in Hospitalized COVID-19 Patients

**DOI:** 10.7759/cureus.14942

**Published:** 2021-05-10

**Authors:** Shayan Iqbal Khan, FNU Versha, Pooja Bai, Parkash Bachani, Muhammad Umair Nawaz, Love Kumar, Sidra Naz, Maha Jahangir, Faizan Shaukat

**Affiliations:** 1 Internal Medicine, Liaquat University of Medical and Health Sciences, Jamshoro, PAK; 2 Medicine and Surgery, Liaquat University of Medical and Health Sciences, Jamshoro, PAK; 3 Internal Medicine, Jinnah Sindh Medical University, Karachi, PAK; 4 Internal Medicine, University of Health Sciences (UHS), Lahore, PAK; 5 Anesthesiology, Dow University of Health Sciences, Karachi, PAK; 6 Internal Medicine, Dow University of Health Sciences, Karachi, PAK

**Keywords:** covid-19, ophthalmological manifestation, corona virus disease, pakistan, clinical characteristics

## Abstract

Introduction: The symptoms of coronavirus disease-19 (COVID-19) may range from mild to severe. Patients usually present with fever, cough, and other respiratory tract symptoms, but may also be asymptomatic. Some studies have also indicated the ocular involvement by the virus. This study aims to look deeply into all ophthalmic findings seen in COVID-19 patients and their clinical characteristics.

Methods: This longitudinal study was conducted in the COVID-19 unit of a tertiary care hospital, Pakistan. Data of patients hospitalized with COVID-19 infection between July 2020 and March 2021 were included in the study. Ophthalmological examination was done at the time of admission and was repeated every alternate day to look for any ophthalmological manifestation.

Results: Out of 441 (n= 441), 61 (13.8%) participants had ophthalmological findings on examination. Patients with ophthalmological findings were significantly younger compared to patients without ophthalmological findings (42 ± 6 years vs. 44 ± 7; p-value, 0.03). C-reactive protein (CRP) was also significantly higher in patients with ophthalmological findings (122.2 ± 16.2 vs. 112.8 ± 19.8; p-value, 0.005). The most common ophthalmological finding was conjunctival irritation (50.8%), followed by diplopia (27.8%) and cotton wool spots (27.8%).

Conclusion: Ophthalmological findings are prevalent in patients with COVID-19. In this study, patients with higher CRP levels were associated with ophthalmological findings. It is important to conduct ophthalmological examinations in patients with COVID-19, as they may give a clue about other complications associated with COVID-19.

## Introduction

The coronavirus disease-19 (COVID-19) was declared a public health emergency by the World Health Organization (WHO) in early 2020, after the outbreak of the severe acute respiratory syndrome coronavirus 2 (SARS-CoV-2) in China [1]. The virus spread quickly to involve other parts of the world, leading to a pandemic, which not only affected the global economy but also led to almost 2.9 million deaths worldwide in its first year [1,2].

The symptoms of COVID-19 may range from mild to severe. Patients usually present with fever, cough, shortness of breath, dyspnea, and other respiratory tract symptoms, but may also be asymptomatic. In the more severe cases, patients may develop severe respiratory disease and pneumonia, which may require intensive care assistance and usually have poor recovery rates [3]. This variation in presentation is poorly understood but the individual host response to the virus may have a role in determining the severity of the disease [4].

Apart from the respiratory tract, COVID-19 is also seen to affect other organ systems resulting in gastrointestinal, cardiovascular, neurological, hematological, and other symptoms. Some studies have also indicated the ocular involvement by the virus [5]. The COVID-19 can affect both the ocular surfaces and the deeper neural tissue. Studies have shown the presence of SARS-CoV-2 in the conjunctival swabs [6]. The usual ocular surface symptoms are that of conjunctival irritation like itching, chemosis, tearing, and foreign body sensation. The presence of these symptoms is seen to correlate with disease severity [6-8]. The retinal involvement with abnormal findings on dilated eye examination has also been reported in COVID-19 patients. Usual abnormalities seen on the exam include ischemia with cotton wool spots and pallor, increased tortuosity, and dilation of vessels as well as the presence of flame-shaped hemorrhages [9,10]. This ocular involvement raises concerns regarding disease transmission between physicians and patients during ophthalmic consultation and also highlights the need to consider long-term detrimental effects on the vision, attributed to the retinal changes, seen in these patients. Therefore, this study aims to look deeply into all ophthalmic findings seen in COVID-19 patients and their implication on general health and disease transmission.

## Materials and methods

This longitudinal study was conducted in the COVID-19 unit of a tertiary care hospital, Pakistan. Data of patients hospitalized with COVID-19 infection between July 2020 and March 2021 were included in the study. All subjects gave their informed consent for inclusion before they participated in the study. Ethical board approval was taken before the start of the study. Patients were treated as per local guideline for the treatment of COVID-19 [11]. Patients with diabetes and hypertension were excluded from the study to avoid including pre-existing ophthalmological manifestation.

Ophthalmological examination, including fundoscopy and tonometry, was done at the time of admission and was repeated every alternate day to look for any ophthalmological manifestation till patient discharge or their death. Patients’ age, gender, lab reports, need for ventilation and ophthalmological findings were recorded in a self-structured questionnaire.

The collected data were analyzed using Statistical Package for Social Sciences® software version 23.0 (SPSS; IBM Corp., Armonk, NY, USA). Mean and standard deviation (SD) were calculated for numerical data. Frequency and percentage were calculated for categorical data. Frequencies were compared using Chi-square. Independent t-test and chi-square were used as appropriate. A p-value less than 0.05 meant that there is a difference between the two groups and the null hypothesis is void.

## Results

A total number of patients included in the study were 441 (n= 441). 61 participants had ophthalmological findings on examination. Patients with ophthalmological findings were significantly younger compared to patients without ophthalmological findings (42 ± 6 years vs. 44 ± 7; p-value, 0.03). C-reactive protein (CRP) was also significantly higher in patients with ophthalmological findings (122.2 ± 16.2 vs. 112.8 ± 19.8; p-value, 0.005) (Table 1).

**Table 1:** Clinical and laboratory characteristics in patients with and without ophthalmologic findings BPM: Breath per minute; CRP: C-reactive protein; LDH: Lactate dehydrogenase; SOC: Standard of care. SD: Standard deviation *: significant, **: not significant

Characteristics (Mean + SD)	Patients with ophthalmological findings (n=61)	Patients without ophthalmological findings (n=380)	P-value
Age (in years)	42 ± 6	44 ± 7	0.03*
Respiratory rate (BPM)	29.6 ± 5.2	28.3 ± 4.9	0.04*
CRP (mg/L)	122.2 ± 16.2	112.8 ± 19.8	0.005*
LDH (IU)	304.3 ± 84.2	300.3 ± 88.2	0.7***
Oxygen saturation (%)	85.2 ± 4.2	86.1 ± 4.9	0.17**
Need for ventilation	11 (18.0%)	61 (16.0%)	0.15**
Death	5 (8.1%)	24 (6.3%)	0.07**

The most common ophthalmological finding was conjunctival irritation (50.8%), followed by diplopia (27.8%) and cotton wool spots (27.8%) (Table 2).

**Table 2:** Ophthalmological manifestations in COVID-19 patients

Ophthalmological findings (n=61)	Number of patients (%)
Conjunctival irritation	31 (50.8%)
Diplopia	17 (27.8%)
Cotton wool spots	17 (27.8%)
Increased intraocular pressure	15 (24.5%)
Micro-haemorrhages	12 (19.6%)
Ophthalmoparesis	11 (18.0%)
Papilledema	06 (9.83%)
Infectious keratitis	04 (6.5%)
Subconjunctival haemorrhage	02 (3.2%)

## Discussion

In our study, participants bearing ophthalmological findings were significantly younger than those who did not bear any. Patients with ophthalmological manifestations showed elevated levels of CRP with a higher respiratory rate. Among the ophthalmological findings, conjunctival irritation was the most common, followed by diplopia and cotton wool spots.

A study conducted by Wu et al. showed that patients with ocular signs and symptoms showed higher levels of CRP with symptoms of conjunctivitis, which were also observed in our study [6]. Many studies have reported that inflammatory markers, such as CRP, play a major role in triggering inflammation [12]. In patients with COVID-19, studies have shown that the majority of them report high levels of inflammatory markers [13,14]. CRP starts elevating within 46 hours of the onset of inflammation and has a half-life of 4-7 hours [15]. This is why it can be used to track active inflammation, such as conjunctivitis, in our case. Moreover, CRP status is also directly linked with the width of lung lesions and the severity of clinical symptoms of the COVID-19 patients [16].

Humans are infected by seven types of coronaviruses (CoVs), namely human coronavirus 229E (HCoV-229E), human coronavirus NL63 (HCoV-NL63), human coronavirus OC43 (HCoV-OC43), human coronavirus HKU1 (HCoV-HKU1), middle east respiratory syndrome coronavirus (MERS-CoV), severe acute respiratory syndrome coronavirus (SARS-CoV), and severe acute respiratory syndrome coronavirus- 2 (SARS-CoV-2) [17]. Most of them are known to cause respiratory infections, but they are also reported to affect the gastrointestinal system and ocular tissues [18,19]. Out of these, NL63 and SARS-CoV-2 are believed to affect the ocular system. Between 2000 and 2003, reports of bronchiolitis and conjunctivitis have been noted due to NL63 [20]. Additionally, in 2004, SARS-CoV-RNA was found in tear specimens of three patients, pointing towards the presence of virus in tears [18]. Theories were proposed stating the possibility of the conjunctiva being the exposed site of the attack by the pathogenic droplets; route of infection from the airway tract through the nasolacrimal duct, or hematogenous infection of the lacrimal gland, were put forward [18]. Analyses based on genome and structure show similarity in the receptor binding sites between SARS-CoV and SARS-CoV-2, with alike pathology and epidemiology [21,22]. Therefore, a similar effect on ocular tissues is expected. However, ophthalmic manifestations have been reported by very few people [6,8].To the best of our knowledge, this is the first study from this region that have studied ophthalmological manifestation in COVID-19 patients. Since the study was conducted in one institute, the sample size was might not have been diverse and care should be taking inferring the result to a greater population. Another limitation might be that this study only included hospitalized patients and does not include mild and moderate cases of COVD-19.

The above-stated literature and the findings of our study suggest that patients with ocular manifestations should be dealt with precautions, and screening tests should be done under the supervision of doctors to avoid further complications. Moreover, more clinical studies involving a larger sample size should be concluded to further evaluate and explore ophthalmic presentation in COVID-19 patients.

## Conclusions

Ophthalmological findings are prevalent in patients with COVID-19. In this study, patients with ophthalmological findings are associated with higher CRP. It is important to conduct ophthalmological examinations in patients with COVID-19, as they may give a clue or help prevent other complications associated with COVID-19. This study also suggests that long-term follow-up of patients with COVID-19 should also include an ophthalmological examination.
